# Hemodynamic Investigation of the Effectiveness of a Two Overlapping Flow Diverter Configuration for Cerebral Aneurysm Treatment

**DOI:** 10.3390/bioengineering8100143

**Published:** 2021-10-16

**Authors:** Yuya Uchiyama, Soichiro Fujimura, Hiroyuki Takao, Takashi Suzuki, Motoharu Hayakawa, Toshihiro Ishibashi, Kostadin Karagiozov, Koji Fukudome, Yuichi Murayama, Makoto Yamamoto

**Affiliations:** 1 Graduate School of Mechanical Engineering, Tokyo University of Science, Tokyo 125-8585, Japan; 4519701@ed.tus.ac.jp (Y.U.); takao@jikei.ac.jp (H.T.); 2 Department of Innovation for Medical Information Technology, The Jikei University School of Medicine, Tokyo 105-8461, Japan; s-fujimura@jikei.ac.jp (S.F.); takashi.suzuki@siemens-healthineers.com (T.S.); 3 Department of Mechanical Engineering, Tokyo University of Science, Tokyo 125-8585, Japan; kfukudome@rs.tus.ac.jp; 4 Department of Neurosurgery, The Jikei University School of Medicine, Tokyo 105-8461, Japan; t-ishibashi@jikei.ac.jp (T.I.); kostadinkaragiozov@yahoo.com (K.K.); ymurayama@jikei.ac.jp (Y.M.); 5 Digital Health & SYNGO Department, Siemens Healthcare K.K., Tokyo 141-8644, Japan; 6 Department of Neurosurgery, Fujita Health University, Toyoake 470-1192, Japan; hayakawa@fujita-hu.ac.jp

**Keywords:** flow diverter, computational fluid dynamics, endovascular treatment, double-stenting technique, hemodynamics, cerebral aneurysm

## Abstract

Flow diverters (FDs) are widely employed as endovascular treatment devices for large or wide-neck cerebral aneurysms. Occasionally, overlapped FDs are deployed to enhance the flow diversion effect. In this study, we investigated the hemodynamics of overlapping FDs via computational fluid dynamics (CFD) simulations. We reproduced the arterial geometry of a patient who had experienced the deployment of two overlapping FDs. We utilized two stent patterns, namely the patterns for one FD and two overlapping FDs. We calculated the velocity, mass flow rate, wall shear stress, and pressure loss coefficient as well as their change rates for each pattern relative to the no-FD pattern results. The CFD simulation results indicated that the characteristics of the blood flow inside the aneurysm were minimally affected by the deployment of a single FD; in contrast, the overlapping FD pattern results revealed significant changes in the flow. Further, the velocity at an inspection plane within the aneurysm sac decreased by up to 92.2% and 31.0% in the cases of the overlapping and single FD patterns, respectively, relative to the no-FD pattern. The simulations successfully reproduced the hemodynamics, and the qualitative and quantitative investigations are meaningful with regard to the clinical outcomes of overlapped FD deployment.

## 1. Introduction

Cerebral aneurysm is a common cerebrovascular condition in which part of the cerebral artery deforms. The frequency of the cerebral aneurysms has been reported to be approximately 1–10% for the total population [1,2,3]. Endovascular treatment methods are widely employed to treat cerebral aneurysms because they are minimally invasive. Flow diverters (FDs) were originally developed as endovascular treatment devices; they have been designed to treat large-, giant-, and wide-neck aneurysms because of their relatively high rupture risks and the difficulty of treatment via conventional endovascular coiling techniques [1,4]. FDs consist of densely braided fine metal wires; the deployed mesh prevents partial blood flow from entering the aneurysm sac and subsequently inducing thrombosis in the aneurysm. Although FDs are useful for treating complex aneurysms, the degree of blood flow reduction is not always sufficient to allow thrombosis formation. For example, Puffer et al. [5] reported that 71% of 44 patients had complete occlusion after FD deployment; thus, 29% of patients did not have insufficient thrombosis formation in their aneurysm. Lubicz et al. [6] also reported the long-term results of FD deployment, and 16% of the patients had remnant flow inside the aneurysm, whereas 11% of the patients showed incomplete occlusion. Thus, some neurosurgeons choose to deploy overlapping FDs to enhance flow diversion relative to that achievable via single-FD deployment.

With the continuous advancement of computing technology, computational fluid dynamics (CFD) simulation has become a popular means to investigate pathological aneurysmal characteristics, such as rupture, growth, and their clinical outcomes [7,8,9,10,11]. In particular, CFD simulations are conducted to investigate the relationships between the hemodynamic parameters and the aneurysm characteristics and outcomes.

In this study, we investigated the hemodynamic environment of a patient with first-hand experience with the deployment of two overlapping FDs to treat a large aneurysm. We reconstructed the patient-specific three-dimensional geometry and simulated the deployment of the FDs inside the artery. We examined the FD stent patterns of (1) a single-FD stent and (2) two overlapping FD stents. We applied CFD to simulate the aneurysm characteristics and the outcomes associated with both stent patterns, as well as those in the case of no FD deployment. We then compared the hemodynamic parameters for all stent patterns.

## 2. Materials and Methods

### 2.1. Patient Selection and Inspection Geometry

**Figure 1:**
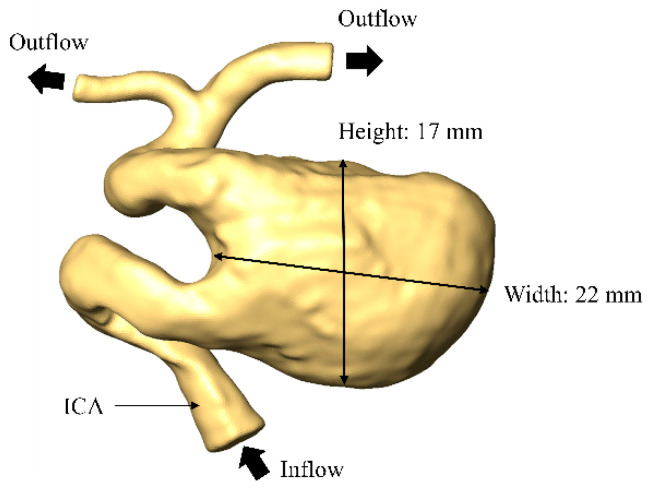
Arterial and aneurysm geometry.

We selected a 78-year-old male patient who had a large aneurysm of the left internal carotid artery (ICA). The aneurysm was 22 mm wide and 17 mm high. The arterial geometry of the patient was obtained by applying four-dimensional digital subtraction angiography. We used the resulting clinical images to reconstruct the patient-specific three-dimensional geometry data for the aneurysm. Then, we generated the surface data and saved them in stereolithography format using the multi-purpose visualization software Amira 5.6 (FEI/VSF-division, Bordeaux, France). Figure 1 shows the reconstructed geometry of the large cerebral aneurysm.

### 2.2. Virtual FD Deployment Methods and Stent Patterns

The patient was treated with two overlapping Pipeline embolization device (Covidien/Medtronic, Minneapolis, MN, USA) stents, which enabled us to model the stent geometry. We applied our in-house virtual stenting program at the Tokyo University of Science in accordance with the methodology suggested by Bouillot et al. [12]. The program reproduces the FD structure based on the following input information: number of wires, wire diameter, length, and stent size. To execute the program, we first reproduced the geometry of the parent artery by excluding the aneurysmal sac and other arterial branches from the original geometry. Then, we calculated the centerline of the parent artery and minimum inscribed sphere radius values corresponding to the centerline by using the Vascular Modeling Toolkit (VMTK, www.vmtk.org; accessed date: 1 August 2021). We subsequently reproduced the FD wires based on the information described above. Regarding the reproduction of the Pipeline device, we referred to the design parameters described by Ma et al. [13]. Additionally, we applied a value of 0.03 mm as the stent wire diameter, as described by Shapiro et al. [14].

**Figure 2:**
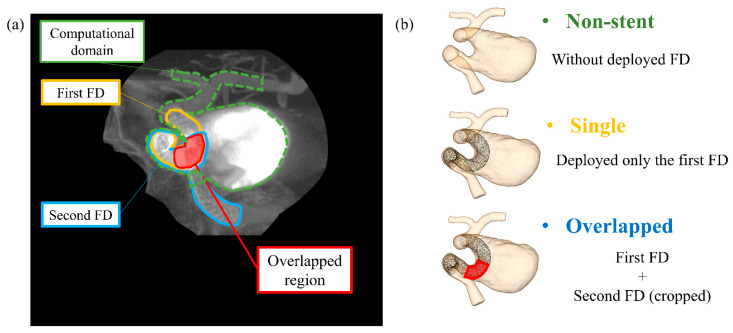
(**a**) Clinical angiography image showing the actual flow diverter (FD) positions and the overlapped region. (**b**) Computational domain for each FD deployment pattern.

We implemented the non-stent pattern as a control pattern, and two stent patterns, i.e., the single and overlapped patterns, and conducted CFD simulations for each pattern. Figure 2 shows (a) a clinical angiography image indicating the two overlapping FD locations and (b) the computational domain for each pattern. It should be noted that we cropped an FD in the overlapped pattern. The second cropped FD corresponds to the clinically deployed location indicated in Figure 2a by a half-toned area. The second FD proximal to the aneurysm was excluded from the FD geometry to reduce the computational cost. The single stent pattern shown in the figure is a reproduction of the surgical conditions associated with aneurysm treatment with only one FD; the overlapped pattern shows the actual conditions for the patient.

### 2.3. CFD Simulation Procedure

We constructed unstructured computational meshes for each of the three stent pattern-specific geometries using ANSYS ICEM CFD 18.1 (ANSYS Inc. Canonsburg, PA, USA). The structure consisted of tetrahedral and prism meshes, where the prism mesh was aligned near the arterial walls and tetrahedral meshes were generated near the FDs and in the other elements. The minimum element size for the FD wires was set to be 0.008 mm, which is equivalent to the wire diameter divided by 12; this scheme was developed by Larrabide et al. [15]. In other areas, the element sizes were set according to the vessel diameters. Under these mesh settings, we achieved mesh convergence and sufficient spatial resolution. The total number of elements was approximately 6 million for the non-stent pattern, 270 million for the single pattern, and 330 million for the overlapped pattern.

The CFD simulations were performed by using the finite volume method solver ANSYS CFX 18.1 (ANSYS Inc. Canonsburg, PA, USA). We assumed blood to be a Newtonian fluid with a density and Newtonian viscosity of 1050 kg/m^3^ and 0.0036 Pa s, respectively. The area of the parent artery proximal to the aneurysm was 1.45× 10^–5^ m^2^, thus its equivalent diameter was 4.30 mm. The average velocity in the artery was 0.217 m/s. Consequently, the Reynolds number was calculated to be 272 in the parent artery. Therefore, we assumed the flow to be incompressible laminar flow. The arterial walls and wires of the FDs were assumed to be rigid. We connected 75-mm-long straight tubes at the inlet and all outlets of each computational domain to reduce the hemodynamic influence of the boundary conditions. Amili et al. [16] showed that the flow pattern is dependent on the aneurysm geometry, especially in large aneurysms that have been deemed eligible for FD deployment based on the results of an in vitro study. Thus, we conducted steady flow simulations for each deployed FD pattern. The inlet condition was a steady flow rate at the ICA of 0.003465 kg/s, as firstly reported by Ford et al. [17]. In addition, the outflow condition entailed fixing the average static pressure to 0 Pa at all outlets [7].

### 2.4. Hemodynamic Parameters and Evaluation

To begin, we investigated the flow structure and characteristics for each simulation pattern; then, we compared the flow characteristics obtained via the CFD simulations to the corresponding two-dimensional angiographic images. We had two images that were taken preoperatively and postoperatively, and these images corresponded to the non-stent and overlapped patterns, respectively. We validated our CFD simulation results by comparing them to the actual imaged blood flow state. Then, we evaluated the hemodynamic parameters as described below.

**Figure 3:**
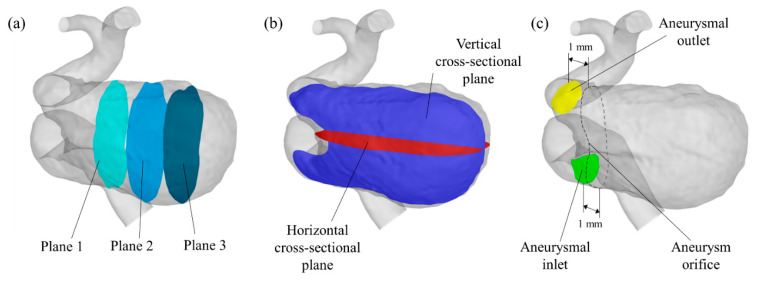
Plane locations for (**a**) flow evaluation, (**b**) flow visualization, and (**c**) parameter calculation.

In previous studies, it was common to investigate the reduction rates of hemodynamic parameters, such as the flow rate, velocity, and wall shear stress (WSS), to establish the relationship between the clinical outcome and hemodynamics [18,19,20,21]. However, to compare the parameters at different locations in each pattern, we constructed three perpendicular planes in the aneurysm sac. These planes were named “Plane 1”, “Plane 2”, and “Plane 3” in order of smallest distance from the parent artery. We also created horizontal and vertical cross-sectional planes in the aneurysmal sac to visualize the flow behavior inside the aneurysm under the conditions of each pattern. To calculate the hemodynamic parameters described below, we constructed two cross-sectional planes that we defined as the aneurysmal inlet and outlet, which were respectively positioned at 1 mm distal and proximal to the aneurysm orifice. The location of each plane is shown in Figure 3.

First, we calculated the area-averaged velocity magnitude $v_{i}$ at Plane *i* (i.e., Planes 1–3), by using the equation below:

(1) $$v_{i}=\frac{\sum_{n}u_{n}S_{n}}{\sum_{n}S_{n}}$$

where n is the number of cells included in the plane, $u_{n}$ is the velocity magnitude for each cell, and $S_{n}$ is the area of each cell. After we calculated the area-averaged velocity magnitude, we calculated the velocity reduction rate by using the following equation:

(2) $$VelocityReductionRate=\frac{v_{Non,i}-v_{i}}{v_{Non,i}},$$

where $v_{Non,i}$ is the area-averaged velocity magnitude for the non-stent pattern at Plane *i*. In addition to the velocity at these surfaces, we evaluated the mass flow rate (MFR) entering the aneurysm. The MFR is the mass flow entering the aneurysmal sac at the aneurysmal orifice, as normalized by the mass flow value at the aneurysmal inlet. Next, we calculated the WSS by using the following equation:

(3) $$\mathrm{WSS}=\mu \frac{du}{dr}$$

where μ is the viscosity, u is the velocity, and r is the distance from the wall to the location at which the velocity u exists. We calculated the area-averaged WSS value for the entire aneurysmal sac for each pattern and compared the calculated values for the stent and non-stent pattern. Finally, we calculated the pressure loss coefficient (PLc), as suggested by Takao et al. [7]. The *PLc* is defined as follows:

(4) $$PLc=\frac{\left(\frac{1}{2}\rho v_{in}^{2}+P_{in}\right)-\left(\frac{1}{2}\rho v_{out}^{2}+P_{out}\right)}{\frac{1}{2}\rho v_{out}^{2}},$$

where ρ is the blood density, and $v_{in}$ and $v_{out}$ and $P_{in}$ and $P_{out}$ denote the average velocity and static pressure at the aneurysmal inlet and outlet, respectively. PLc characterizes the contribution of the artery and FD wires to energy dissipation.

Moreover, we calculated the reduction rates for the MFR, WSS, and PLc in the same manner as that described for velocity (see Equation (1)).

## 3. Results

### 3.1. Blood Flow Characteristics in the Aneurysm

**Figure 4:**
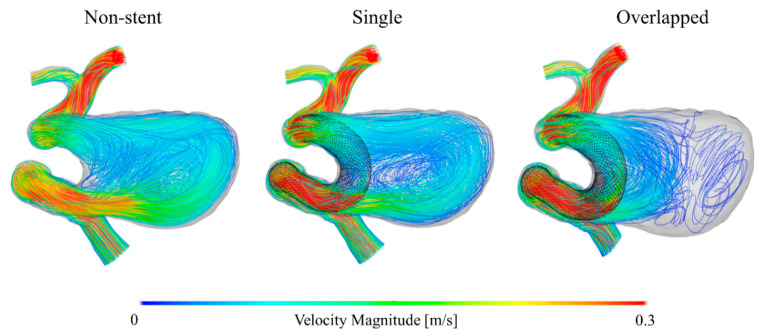
Blood flow visualization for each stent pattern using colored streamlines.

**Figure 5:**
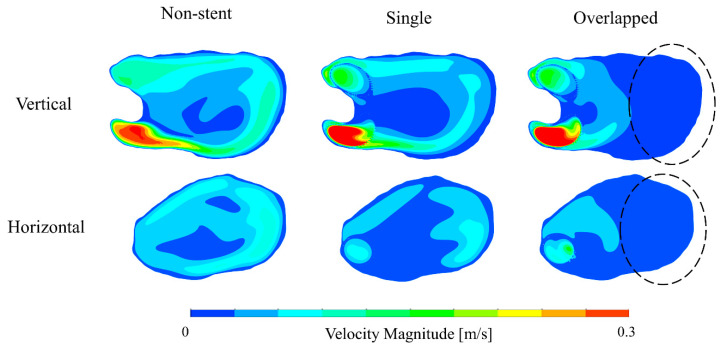
Blood flow velocity map results for vertical and horizontal cross-sectional planes.

**Figure 6:**
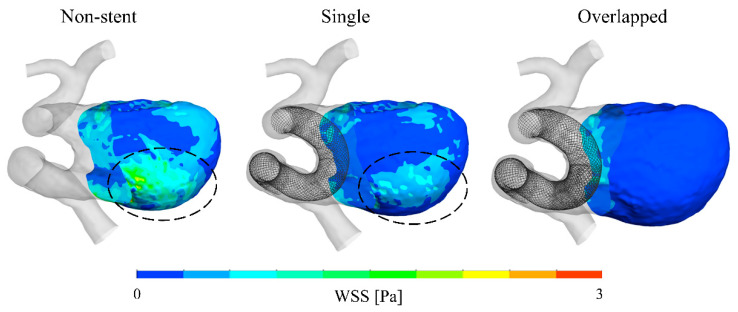
Wall shear stress (WSS) map results for the aneurysmal sac corresponding to each pattern.

To compare the flow structures qualitatively, we visualized the blood flow using streamlines, as shown in Figure 4. The single pattern yielded a flow pattern somewhat similar to that of the non-stent pattern, i.e., both patterns produced blood flow patterns with characteristic large swirls in the aneurysmal sac. In contrast, the overlapped pattern generated a different flow structure, showing that the flow stagnated within the aneurysmal sac. Furthermore, the extent of the decrease in flow velocity was considerably larger in the case of the overlapped pattern than in the case of the single pattern. Figure 5 shows the velocity maps corresponding to the vertical and horizontal cross-sectional planes for each pattern. In the cases of the non-stent and single patterns, blood flowed into the aneurysmal sac in the lateral direction and flowed along the aneurysm wall. However, the flow seemed to enter the sac in an inclined direction before being redirected to the outlet in the overlapped pattern. Additionally, the flow was stagnated at the center of the aneurysmal sac in the case of the single pattern; however, the flow stagnated over roughly half of the area of the aneurysmal sac in the case of the overlapped pattern, as denoted by the dashed line circles in Figure 5. Lastly, Figure 6 shows the WSS map results for each case. Because the blood flow entered the aneurysmal sac in the lateral direction and flowed along the aneurysm wall in the cases of the non-stent and the single patterns, the WSS was relatively high near the bottom of the aneurysmal sac. In contrast, the WSS tended to be low in the case of the overlapped pattern because of flow stagnation.

### 3.2. Validation of CFD Simulation Results: Comparison to Clinical Images

**Figure 7:**
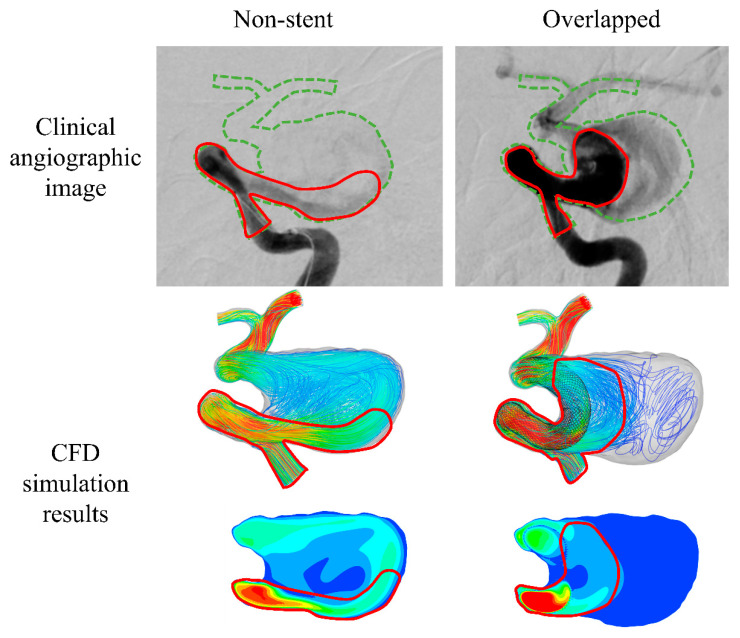
Clinical angiographic images and computational fluid dynamics (CFD) simulation results. The red lines in the angiographic images indicate the visible contrast medium flow.

For comparison, the clinical angiographic images are shown in Figure 7 along with our simulated results. In the figure, the main flow of the injected contrast medium is indicated by red lines. In the case of the non-stent pattern, the contrast medium entered the aneurysmal sac in the lateral direction. This tendency is also observed in the corresponding CFD simulation results. Moreover, in the case of the overlapped pattern, the contrast medium did not flow in the lateral direction into the aneurysmal sac. This flow tendency is similar to that observed in the corresponding simulated results. Unfortunately, we were unable to obtain a clinical angiographic image for the single-FD case. Nevertheless, we observed good consistency between the simulated results and the corresponding actual conditions in both the non-stent and overlapped patterns.

### 3.3. Quantitative Hemodynamic Evaluation

**Figure 8:**
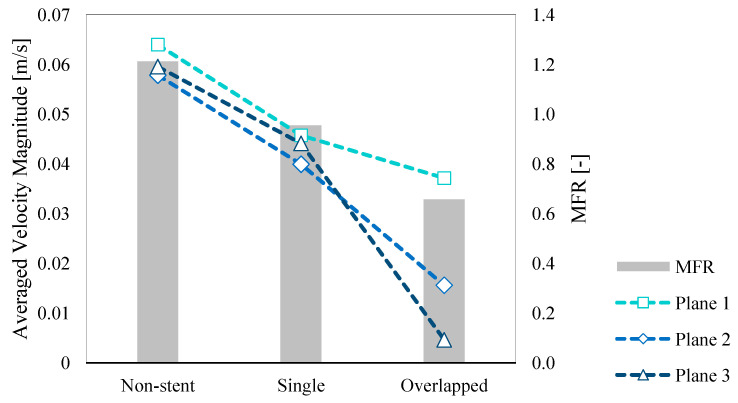
Calculated average velocity magnitude and mass flow rate (MFR) results.

We calculated the average velocity magnitudes at Planes 1, 2, and 3 for each pattern and MFR; the results are summarized in Table 1 and illustrated in Figure 8. We briefly discussed the similarity between the flow structures associated with the non-stent and single patterns in the previous section. This trend is also observed in the case of the average velocity magnitude (Figure 8), in that there is similarity for all three planes; specifically, the value for Plane 2 is lower than those for the other two planes. The overlapped pattern shows a different trend, as can be seen in Figure 8; specifically, the flow velocity at Plane 3 is lower than the velocity at the other planes. Additionally, for the stent patterns, the trend of the MFR decrease is similar to that observed for the velocity.

**Figure 9:**
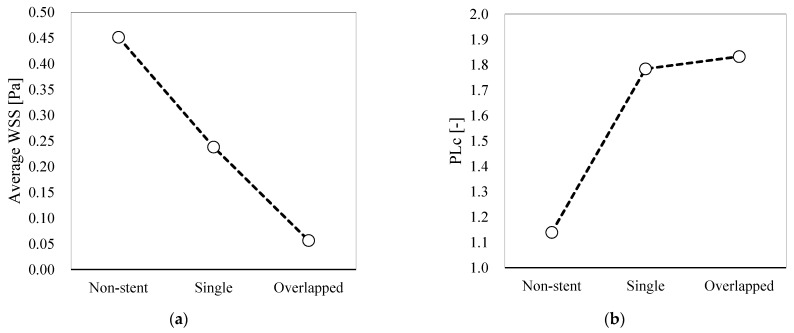
(**a**) Average WSS for each pattern and (**b**) pressure loss coefficient (PLc) values for each pattern.

In addition, the WSS for each pattern, as averaged over the entire aneurysmal sac, is shown in Figure 9a, and the calculated PLc values are presented in Figure 9b. In the case of the single pattern, the WSS is nearly half that observed in the case of the non-stent pattern. The WSS is even lower in the case of the overlapped pattern. Additionally, the PLc associated with the single pattern is considerably larger than that associated with the non-stent pattern. However, the difference between the PLc values associated with the single and overlapped patterns is slight.

According to Equation (1), the single pattern results reveal a 28.5% velocity reduction rate at Plane 1. We calculated the velocity reduction rates for the other inspection planes, and the results are summarized in Table 2. The single-pattern velocity reduction rate is approximately 30% at the inspection planes; however, this rate is higher for the overlapped pattern, reaching 92.2% at Plane 3 because of flow stagnation. This trend is also observed for the MFR. Specifically, the reduction rates for the single and overlapped patterns are 21.3% and 45.8%, respectively. Similarly, the single-pattern reduction rate for the WSS is 47.3%, whereas the reduction rate is 87.5% in the case of the overlapped pattern. As shown in Figure 9b, the PLc is higher because of the FD deployment; thus, the reduction rate is shown as negative in Table 2. The difference between the single- and overlapped-pattern PLc reduction rates is 4.26%, which is low as compared with the differences observed for the other hemodynamic parameters.

## 4. Discussion

### 4.1. Agreement between Simulated Results and Angiographic Images

Angiography generally has good spatial resolution, and it enables visualization of the flow status via observation of the contrast medium movement within the blood stream. In previous studies, the flow structures obtained via CFD simulation have been compared with the flow states determined from angiograms. For instance, Cebral et al. [22] developed visualization methods that enabled virtual angiography and then analyzed the corresponding CFD simulation results. The simulated CFD results showed good agreement with the clinical angiographic images. In addition, Mut et al. [23] conducted CFD simulations of aneurysms with an intrasaccular device referred to as the Woven EndoBridge aneurysm embolization device (Sequent Medical, Aliso Viejo, CA, USA). They reported that the simulated results were in good qualitative agreement with angiographic images. However, to the best of our knowledge, there are no similar studies of the influence of the FD status on the flow status. Thus, this study may be the first time that the flow structure has been investigated according to the FD status, as well as the first time that simulated results that show consistency with the corresponding angiographic images have been obtained. As such, we believe that our CFD simulations could reproduce the actual hemodynamic environment of the aneurysm with reasonably accuracy. Although we validated the simulated results qualitatively, we did not do so quantitatively. Such tasks remain to be considered in future studies.

### 4.2. Hemodynamic Differences between Single and Overlapped Stent Patterns

#### 4.2.1. Velocity and MFR

In the case of FD deployment, the treatment goal is to induce thrombosis in the aneurysmal sac and consequently occlude it. In previous studies, the rate of velocity reduction was investigated because it is believed to be related to thrombosis formation [24]. As shown in Figure 8 and Table 2, the overlapped pattern achieved a considerably higher velocity reduction rate (reaching 92.2%) than the single pattern (31.0%). Thus, the overlapping FD stent configuration seems to be a superior clinical alternative to single-FD deployment in terms of its therapeutic effect as a treatment for complex aneurysms.

Notably, the reduction rates for the single pattern are close to 30%. This finding may indicate that, in the case of the single pattern, the blood flow inside the aneurysm was suppressed for the whole of the aneurysm as a result of the first FD deployment. These results imply that, although the flow structure associated with the single pattern was suppressed to some extent, the qualitative and quantitative trends were remarkably similar to those associated with the non-stent pattern. Alternatively, the overlapped pattern achieved a much higher reduction rate at Plane 3 (i.e., 92.2%). The single pattern could only achieve a corresponding reduction of only 25.9%, i.e., a difference of 66.3%. Thus, the overlapping FD configuration yielded a significantly stronger effect on the hemodynamics than the single-FD configuration.

The overlapped pattern could also achieve a higher MFR reduction rate than the single pattern. Additionally, although the overall trend of a decrease seemed to be similar to that of the velocity, the value for the MFR reduction rate achieved by the overlapped pattern was not as high as the value for the velocity at Plane 3. Conversely, the trends were rather similar at Plane 1, because the inflow conditions were the same for all simulation patterns, ensuring that the mass flow at the aneurysmal inlet would be the same. Thus, the differences in the MFRs reflected the mass flow at the aneurysmal orifice that was close to Plane 1.

#### 4.2.2. WSS

The average WSS calculated for the overlapped pattern was 87.5% lower than that observed for the non-stent pattern. Interestingly, although the flow structures associated with the single stent and non-stent patterns were somewhat similar, the WSS associated with the single pattern was lower by 47.3%, corresponding to a 40.2% difference between the single- and overlapped-pattern values. This difference is attributable to the flow separation and flow stagnation for the overlapped pattern. The WSS is believed to be an important hemodynamic parameter, as it is associated with aneurysmal pathologies, such as initiation, growth, and rupture [25,26,27]. In addition to having a relation to these pathologies, a reduction in WSS is believed to lead to aneurysmal thrombosis formation and successful clinical outcomes [28,29,30]. Thus, our WSS results suggest that, although flow modification and a therapeutic effect can be achieved with only one FD, the overlapping FD configuration can reduce WSS more effectively.

#### 4.2.3. PLc

Although the pattern-specific trends observed in the flow velocity and WSS results were similar, this was not the case for the PLc results. Deployment of the single FD resulted in a 56.6% increase in the PLc value relative to that calculated for the non-stent pattern. However, there was only a slight difference between the single and the overlapped pattern PLc values (i.e., 4.26%). As mentioned in Section 2.4, the PLc provides information concerning the energy dissipation due to the arterial walls and FD wires. Thus, the difference between the single- and non-stent-pattern PLc values is a direct indicator of the energy expenditure of the single-FD geometry. However, the introduction of the second FD can be presumed to have only minimally affected the energy dissipation, because the PLc value for the overlapped pattern was not much different from the value for the single pattern. Nevertheless, the second FD seemed to change the flow direction, rather than suppress the flow. The locally dense stent mesh of the overlapped pattern served to alter the direction of entering the main blood flow from the horizontal to inclined angle without flow energy suppression; this approach shifted the main flow away from the aneurysm wall and may be the reason why the overlapped pattern was found to generate a large stagnant region.

### 4.3. Locally Dense FD Mesh and Its Beneficial Effects on the Hemodynamic Environment

**Figure 10:**
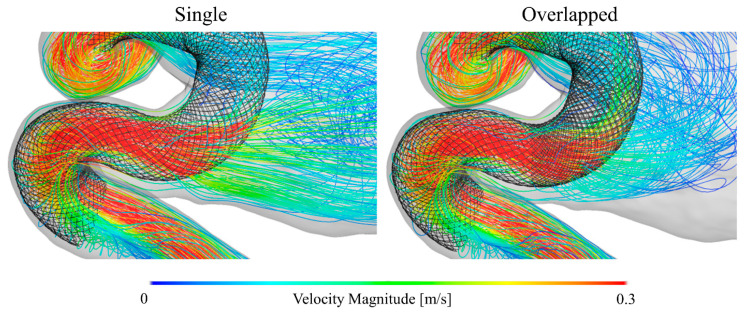
Blood flow entering the aneurysmal sac in the presence of the single or the overlapped pattern.

The differences between the hemodynamic parameters of the single and overlapped patterns seem to be associated with the wire mesh density of the FDs. As shown in Figure 2b, the deployed FDs are bent so as to fit in the carotid artery “siphon”, and the FD geometry is accordingly deformed. This bending-induced FD deformation resulted in mesh expansion, the consequence of which was a reduced mesh density along the outer (convex) side of the bend. Figure 10 shows enlarged views of the main flow entering the aneurysmal sac under the conditions of single and overlapped pattern deployment. The flow entering the aneurysmal sac had to pass through the expanded FD region, which weakened the flow diversion effect. Consequently, the flow velocity associated with the single pattern is slightly lower than that of the non-stent pattern. However, in the case of the overlapped pattern, the second FD could cover the expanded region, reinforcing the less dense FD mesh in the first FD. This characteristic can explain why the extent of velocity reduction was larger for the overlapped pattern than the single pattern. The second FD also covered the aneurysm inflow section, which may have also contributed to the larger velocity reduction. These results imply that the flow diversion effect may be influenced by arterial geometry and that the overlapping FD stent configuration can effectively compensate for localized low-density mesh areas.

There are methods to increase the local mesh density of FDs that involve overlapping more than one FD or compacting the FD, which is referred to as the push-pull technique [31,32,33,34]. Our results may provide guidance regarding where the FDs should be overlapped or compacted to maximize the flow-diverting effect. In this study, we conducted CFD simulations that reproduced the hemodynamic environment within the ICA of a patient with two overlapping FDs. The simulated results provide insight into how the introduction of a second FD near the aneurysm inlet section can strengthen the flow diversion effect more than the deployment of a single FD. The blood flow velocity within the section was relatively high compared to that within the other regions inside the aneurysmal sac, as can be seen in Figure 4 and Figure 10. Thus, we believe that it is important to cover the overlapping FD or compact its structure in the region near the aneurysm inflow section. A clear understanding of the flow velocity reduction and flow direction alteration associated with FD deployment is necessary to optimize the hemodynamic environment in the aneurysm.

The local dense FD mesh quantitively reduces the flow in this case; however, the overlapping FDs occasionally have negative effects. The treatment concept of FD deployment involves inducing thrombosis inside the aneurysm. However, it has been reported that the overlapped FDs have a higher risk of in-stent thrombosis [35]. The thrombus may occlude the distal artery; thus, the local dense mesh has both a higher flow reduction effect and risk of complication. It is important to accomplish high effectiveness with the minimum risk of complication. CFD simulations may provide insight into an optimized FD configuration that has a high therapeutic effect and low complication risk.

### 4.4. Limitations and Future Work

Some investigations have been conducted on hemodynamics with several FD deploy configurations. Damiano et al. [31] investigated the hemodynamics in three aneurysms with three different FD configurations (single, two overlapping, and single compacted). However, these aneurysms were representative cases, and these FD configurations did not correspond to actual clinical strategies. In this study, in contrast, we investigated the hemodynamics in an aneurysm that was treated with two overlapped FDs. Unfortunately, we were unable to obtain any clinical information regarding the treatment outcome and, specifically, whether the aneurysm was successfully occluded. To determine whether overlapping FD deployment yields better clinical outcomes, a study should be conducted with a different design and using a different patient dataset. We found that deploying the second FD just over the aneurysm inlet section increased the local mesh density, leading to a larger velocity reduction. Additional studies must be conducted to improve our understanding of the conditions surrounding overlapping FD deployment. For example, future studies should focus on determining the combination of deployed FDs that would most effectively optimize the velocity reduction and flow diversion. Furthermore, in this study, we did not focus on optimizing the positioning angle of the overlapped FDs, which may affect the local mesh density.

We assumed the blood flow acts as a Newtonian fluid for simplicity, although actual blood is a non-Newtonian fluid with a viscosity that depends on the shear rate, which is strongly influenced by biologically related reactions [36,37]. Gambaruto et al. [38] applied non-Newtonian blood viscosity model assumptions in their calculations and reported that the WSS can be influenced by the viscosity model. Further, Morales et al. [39] concluded that the non-Newtonian model is not necessary for conducting CFD simulations of coiled aneurysms owing to the minimal influence of the variable viscosity on the flow structure within the aneurysmal sac. However, in the cases of the FD-treated aneurysms, the relationship between the non-Newtonian viscosity model and hemodynamic environment remains unclear; thus, it should be investigated in future studies.

In addition to the fluid model, it is necessary to consider the simulation parameters, such as the inflow and outflow boundary conditions, and wall assumption. In this study, we applied the flow rate reported by Ford et al. [17] as the inflow boundary condition; however, a more realistic reproduction of the aneurysm hemodynamics mandates the application of patient-specific conditions. The flow velocity parameters for these conditions can be derived from patient clinical images, such as magnetic resonance images or angiograms [40,41]. Additionally, considering that the outflow condition is believed to influence the local hemodynamics, the related parameters should also be patient specific [42,43,44]. Notably, although the vessel wall has viscoelastic characteristics, we assumed it to be rigid. Furthermore, the relationships between the mechanical properties of the wall and the hemodynamic environment remain unclear. We may investigate these relationships by conducting fluid–structure interaction simulations. It is necessary to optimize our simulation criteria by eliminating these limitations; this will enable more accurate and realistic reproduction of the hemodynamic environments associated with aneurysmal pathology and the corresponding clinical outcomes.

## 5. Conclusions

We investigated the hemodynamic environment of a cerebral aneurysm that was treated with two overlapped FDs. We applied CFD to simulate the non-treatment of an aneurysm, as well as the treatment of an aneurysm with a single or an overlapping FD deployment pattern. The flow structure observed under the single-FD pattern conditions was similar to that observed when no FD was deployed. However, the simulation of the two overlapping FDs indicated significantly different flow behavior from that associated with the other two simulated patterns. In addition, the overlapping FD pattern results revealed substantial reductions in flow velocity and WSS relative to the non-stent case. Our simulation successfully reproduced the hemodynamic environment of an aneurysm, and the qualitative and quantitative investigations are anticipated to be meaningful for cerebral aneurysms and FD deployment.

## Figures and Tables

**Table 1:** Average velocity magnitude results at the inspection planes for each pattern.

Parameters		Stent Patterns		
		Non-Stent	Single	Overlapped
Average Velocity Magnitude [×10^–3^ m/s]	Plane 1	64.0	45.7	37.1
	Plane 2	57.8	39.9	15.6
	Plane 3	59.6	44.1	4.62
MFR [-]		1.21	0.954	0.657

**Table 2:** Calculated velocity reduction rates for each pattern.

**Parameters**		**Reduction Rate [%]**	
		**Single**	**Overlapped**
Velocity	Plane 1	28.5	41.9
	Plane 2	31.0	73.1
	Plane 3	25.9	92.2
MFR		21.3	45.8
WSS		47.3	87.5
PLc		−56.6	−60.9
